# Radiation Oncology Training in Poland: Multi-institutional Survey

**DOI:** 10.1007/s13187-020-01702-8

**Published:** 2020-02-12

**Authors:** Aleksandra Napieralska, Bartłomiej Tomasik, Mateusz Spałek, Artur Chyrek, Jacek Fijuth

**Affiliations:** 1 Radiotherapy Department, Maria Skłodowska-Curie Cancer Centre, ul. Wybrzeże Armii Krajowej 15, 44-101 Gliwice, Poland; 2 Department of Biostatistics and Translational Medicine, Medical University of Lodz, Lodz, Poland; 3 Department of Radiation Oncology, Medical University of Lodz, Lodz, Poland; 4 Postgraduate School of Molecular Medicine, Medical University of Warsaw, Warsaw, Poland; 5 Department of Soft Tissue/Bone Sarcoma and Melanoma, Maria Skłodowska-Curie Institute - Oncology Center, Warsaw, Poland; 6 Brachytherapy Department, Greater Poland Cancer Centre, Poznań, Poland

**Keywords:** Education, Training quality, Radiation oncology, Residents

## Abstract

To evaluate the quality of radiation oncology training in Poland and to identify difficulties and needs of radiation oncology trainees (ROTs), an anonymous online survey using Google Forms was conducted in November 2018. All ROTs from Poland (*n* = 154) were invited to complete the survey. The survey consisted of 30 questions and regarded aspects such as satisfaction from training, cooperation with the supervisor, and education. During the study period, 105 ROTs from 22 Polish cities responded (the response rate was 68%). Almost 85% were satisfied with the choice of specialty, 43% with training and 81% with the teaching place. Clinical cooperation with the supervisor was described as difficult by 25%. With education, 38% of responders experienced obstacles and another 25% stated that educational cooperation with their supervisor was none. “Good practice” understood as treatment according to the latest guidelines or internal protocols were reported by 71%. Obligatory traineeships were shorter than recommended according to 67% of the responders, usually due to shortage of medical staff. Almost two-thirds of ROTs work only in clinics, while 34% perform both clinical and scientific work. The responders suggested that the introduction of organ-specified courses is an appropriate modification of the current program. Noteworthy, current diagnostic imaging and physics courses are not sufficient for the majority of participants (81% and 80%, respectively). Radiation oncology training is conducted relatively well in Poland. Some hospitals need to improve clinical and educational cooperation between trainees and their supervisors.

**Electronic supplementary material:**

The online version of this article (10.1007/s13187-020-01702-8) contains supplementary material, which is available to authorized users.

## Introduction

Polish radiation oncology training (residency) lasts 5 years. During this 5-year period, residents gain clinical experience under supervision and take part in educational courses and internships. The training finishes with written and oral exams evaluating the knowledge of the trainee (board exam). The topic of the radiation oncology teaching programme and its quality has been an issue of a debate for many years; however, there is no consensus regarding the best methodologies for such evaluation.

The first project of the European Society for Radiotherapy and Oncology (ESTRO) relating to the issue of radiation oncology training was published in 1991 [1]. After that time, another three updated versions of this core curricula were published in 2004, 2007 and in 2011 [2, 3]. This topic was also a matter of a debate in several publications regarding national survey results concerning trainings held in France, Germany, the UK, Italy, the USA and Canada [4–11]. One Polish nationwide survey-based evaluation of the training was performed in 2007. However, it did not contain detailed questions regarding several aspects of specialty programme [12]. In 2017, Benstead K. et al. published multinational survey results which included an opinion of a specialist in medical oncology, radiation oncology and clinical oncology and referred to variations in training and multidisciplinarity across Europe [13].

In 2018, an international survey conducted by ESTRO evaluated responses from 34 countries, but Polish responders were underrepresented in that study [14]. What was also mentioned by Bibault JE et al. is the fact that currently each nation defines the knowledge and enumerates skills required to become a radiation oncologist; thus, separate evaluation is needed for each country [14]. The majority of aforementioned analyses were made by so called young sections of the nations, which was justified by the fact that radiation oncology trainees (ROTs) are the primary source of retroactive information on the teaching system. In 2018, the Polish Society of Radiation Oncology (Polish abbreviation – PTRO) formed a youngPTRO section. One of the first aims of the newly formed group was to address the issue of training quality and to identify difficulties and needs in ROTs in Poland. Thus, at the end of 2018, a large nationwide survey was carried out and its results are presented in this study.

## Methods

An anonymous survey was conducted online using the Google Forms (https://docs.google.com/forms/). The survey was available from 18th October to 9th November 2018. All ROTs (*n* = 154) from Poland were invited to do the survey. Participants were invited via social media (Facebook), e-mail and personal messages. One follow-up reminder message was sent during the study period. Participants were allowed to answer the survey only once.

### Survey Description

A group of Polish senior ROTs constructed a survey, including several aspects of training: satisfaction from training, cooperation with a supervisor, and education (courses, traineeships, conferences, journal clubs and engagement in scientific work). The survey consisted of 30 questions, 9 of which were constructed as the 5-point Likert scale. Two were open questions and the others were single- or multiple-choice questions. No validation testing of the questionnaire was performed. In order to check whether questions were neutral and easy to understand, a sample questionnaire was administered to five senior ROTs. The format was consequently modified following their suggestions. The translated version of the survey is available in Supplementary File 1.

### Statistical Analysis

The survey results are mostly descriptive. Comparative statistics were very limited because of the relatively small sample size. Statistics were calculated using Microsoft Excel® and Statistica 13.1 (Tulsa, OK, USA) software.

## Results

### Response Rate and Demographic Data

**Fig. 1:**
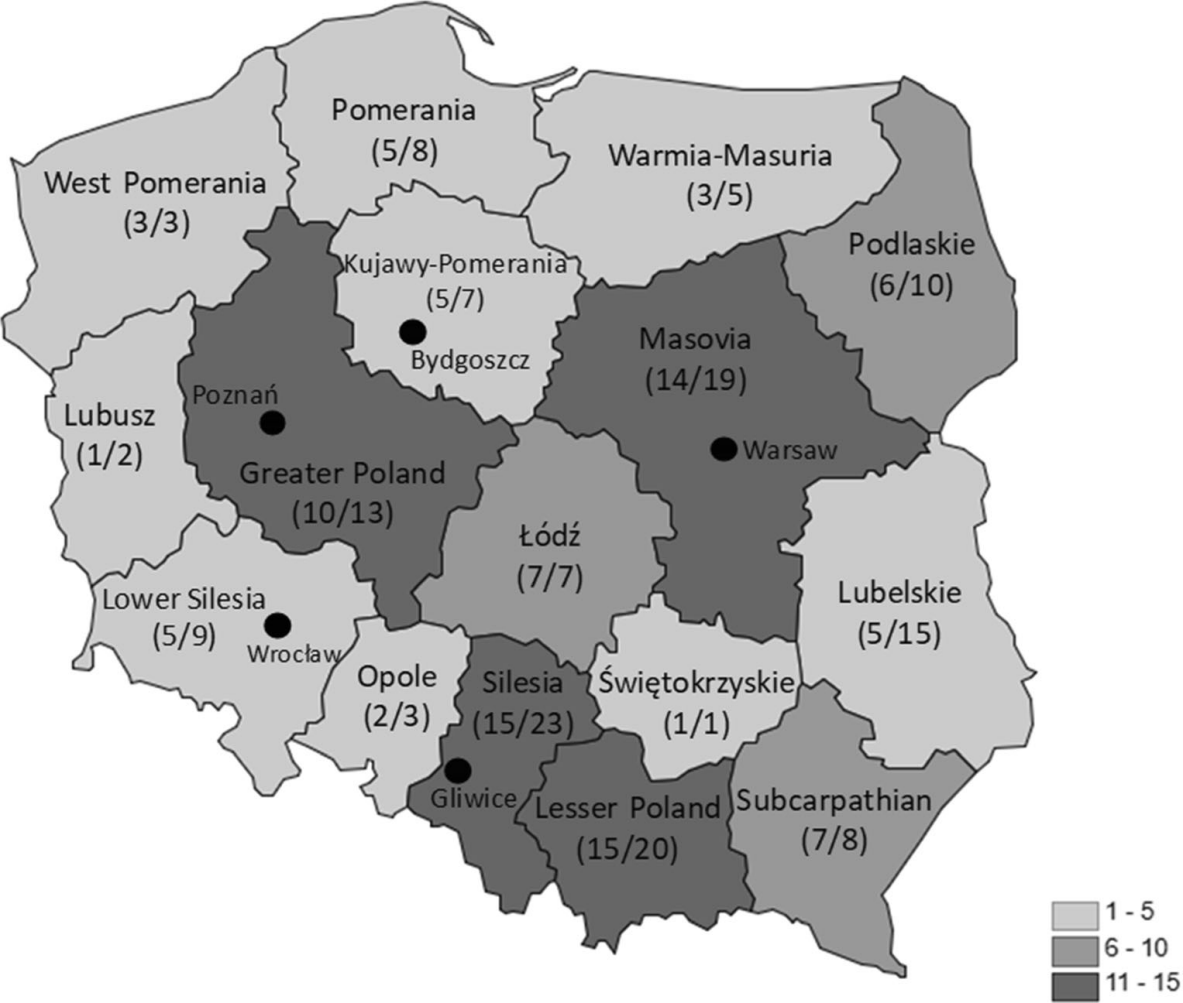
Number of answers received from particular voivodeships and the total number of radiation oncologists in training in each region. Light grey, answers received from 1 to 5 ROTs; medium grey, answers received from 6 to 10 ROTs; and dark grey, answers received from 11 to 15 ROTs

A survey was sent to 154 Polish ROTs. During the study period, 105 of them responded (the response rate was 68%). The responders represented institutions localized in 22 cities in all administrative regions (voivodeships) of Poland (Table 1 and Fig. 1). The majority (67.6%) of responders were trainees of the last 3 years of residency, 18.1% had been working for 1 to 3 years and 14.3% for less than a year.

**Table 1:** Number of participants representing voivodeships of Poland

Voivodeship	Number of respondents	Percentage of total respondents
Lesser Poland	15	14.3
Silesia	15	14.3
Greater Poland	10	9.5
West Pomerania	3	2.9
Subcarpathian	7	6.7
Łódź	7	6.7
Lubusz	1	0.9
Mazovian	14	13.3
Podlaskie	6	5.7
Świętokrzyskie	1	0.9
Opole	2	1.9
Kujawy-Pomerania	5	4.8
Lower Silesia	5	4.8
Warmia-Masuria	3	2.9
Lubelskie	5	4.8
Pomerania	5	4.8
Not indicated	1	0.9
Total	105	100%

### Satisfaction from Training

**Fig. 2:**
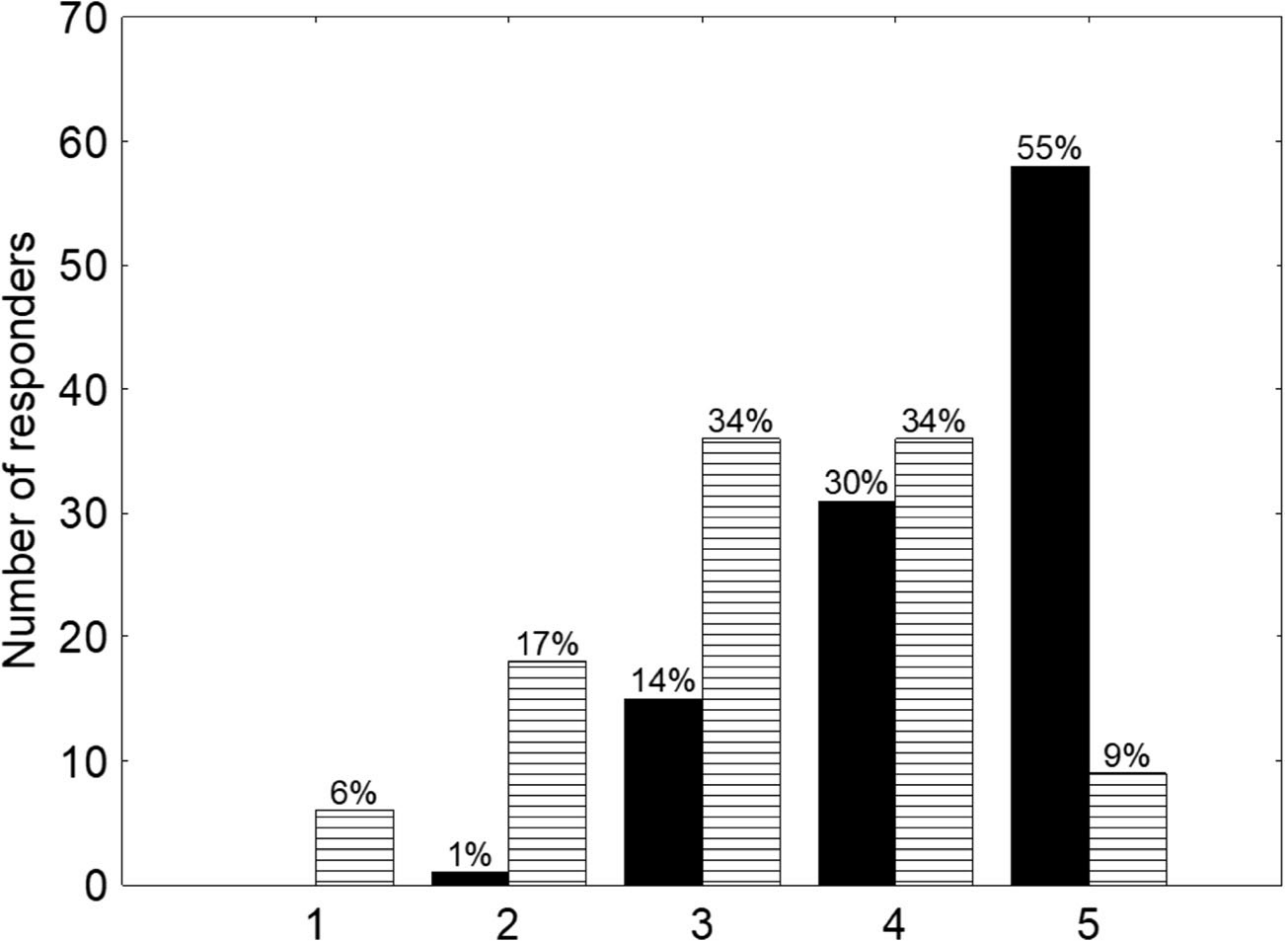
Satisfaction with the choice of specialty in radiation oncology and with the quality of training in radiation oncology. A 5-point scale was used to categorize qualitative answers (from 1, which stands for less satisfied to 5, which stands for very satisfied). Black-coloured columns represent answers regarding satisfaction with the choice of specialization in radiation oncology, while white stripe columns represent answers concerning satisfaction with the quality of the training

The vast majority (85%) of participants were satisfied with the choice of radiation oncology as a specialty (4 or 5 points on the Linkert scale), while only 43% were satisfied with training (Fig. 2).

The majority of responders (81%) were satisfied with the choice of hospital in which training is held, although 55 (52.4%) of them think that they spend too much time at work. When asked about the number of patients treated every month, 6.7% responded that they irradiate less than ten patients per month, 52.4% irradiate ten to twenty patients per month, 25.7% irradiate twenty to thirty patients per month and 15.2% irradiate more than thirty patients every month. Slightly more than half of the responders (52.4%) think that the number of treated patients is adequate, while 16.2% answered that the number of patients is too small. About 18.1% claimed that they have to treat too many patients. Among them, 18.1% responded that the diversity of oncological cases is unsatisfactory.

Independent (without direct supervision) work in the clinic or outpatients’ office is another issue. According to the programme, a radiation oncologist in training is allowed to work as an individual practitioner after the first 2 years of education. Bearing in mind that 32.4% of responders had been trainees for not longer than 3 years, it is not surprising that 28.6% of participants declared that they are not working in the clinic. Interestingly, among 71.4% of the responders who work in the clinic, only 65% do so after finishing 2 years of education. Besides, 54% of responders work also outside the primary place of employment, but the majority (80%) do that due to financial reasons.

### Cooperation with Supervisor

During the whole period of education, a trainee must be supervised by a radiation oncologist who has at least 5 years of experience. In the survey, we asked two questions about clinical and educational cooperation with supervisors. Close, constant clinical cooperation and co-shared patients care with the specialist was declared by 30.5% responders. Almost half (44.8%) of the responders took care of patients independently of a specialist, but cases and treatment plans are consulted by the supervisor. Clinical cooperation was described as difficult by 24.7% of responders, but all of them admitted they were provided help by supervisors or other doctors. Educational cooperation was reported to be very good (regular consultations, discussing the guidelines or latest reports) by 21% of the trainees. About 26% of them stated that the cooperation was good (discussing the guidelines, referring to the literature). What was disturbing, 38% claimed that educational cooperation was difficult (rare conversations about guidelines), whereas 25% of responders complained of complete lack of educational cooperation with their supervisors.

### Education

The programme of specialization in radiation oncology in Poland includes several ways of education: (1) obligatory and recommended courses, (2) traineeships, (3) participation in conferences, (4) participation in journal clubs and (5) scientific work. Four years ago, the programme of specialization changed, but the majority of responders did ‘the older version’, i.e. the one implemented before 1 October 2014.

1. **Courses**

List of mandatory curses is presented in Table 2. Courses are organized in major hospitals and last 3 to 5 days. On the last day of a course, trainees are required to do a test which is aimed to evaluate their knowledge, gained during the course. Courses organized by ESTRO are recommended, but they are not mandatory to finish the specialization.

**Table 2:** List of mandatory courses

List of courses mandatory in years 2003–2014	List of courses mandatory from 01.10.2014 until now
1. Basics of oncology (5 days) 2. Molecular biology, genetic, nuclear medicine and diagnostic imaging (5 days) 3. Basics of medical physics. Radiotherapy and brachytherapy planning (5 days) 4. Experimental and clinical radiobiology, different ways of fractionation (3 days) 5. Standard radiotherapy methods (5 days) 6. Basics of combined treatment of neoplasms and analysis of clinical trials (5 days) 7. Conformal radiotherapy, quality assurance in radiotherapy (3 days) 8. Brachytherapy (5 days) 9. Public health (5 to 10 days) 10. Validation course (5 days) And 2 from 3 below: 11. Head and neck tumours (5 days) 12. Tumours pathology (5 days) 13. Paediatric radiotherapy (5 days)	1. Basics of medical physics and radiobiology (5 days) 2. Diagnostic imaging and nuclear medicine (5 days) 3. Basics of genetics of cancer, pathomorphological diagnostics, combined treatment of neoplasms and analysis of clinical trials (5 days) 4. Radiotherapy and brachytherapy planning (5 days) 5. Head and neck tumours (5 days) 6. Gynaecological and breast cancers (5 days) 7. Thoracic neoplasms (4 days) 8. Gastrointestinal neoplasms (4 days) 9. Genitourinary neoplasms (4 days) 10. Haematological malignancies, TBI (3 days) 11. Central nervous system tumours, paediatric neoplasms, sarcomas, skin cancer and melanoma (5 days) 12. Validation course I: clinics (5 days) 13. Validation course II: radiotherapy planning (5 days) 14. Emergency medicine (5 days) 15. Public health (5 days) and medical jurisdiction (3 days) 17. Medical law (3 days)

Questions referring to the courses showed that public health is the most useless course (reported by 28%). Moreover, some of the introduction courses (like basics of oncology) were conducted within the last years of the training. Responders noticed that the selected lectures were repeated if many courses were held in the same hospital. In the open-form question regarding the most demanded new topics of courses, many suggested these were limited only to organ-specific treatment that is available after updating the programme = in 2014. Responders mentioned also: evidence-based medicine and basics of statistics (*n* = 10), delineation courses (*n* = 26), physics and radiotherapy planning (*n* = 15) and radiographic anatomy (*n* = 18). Some of the responders claimed they could do with internal medicine (*n* = 4), palliative medicine (*n* = 4) and psychooncology (*n* = 3) courses.

**Fig. 3:**
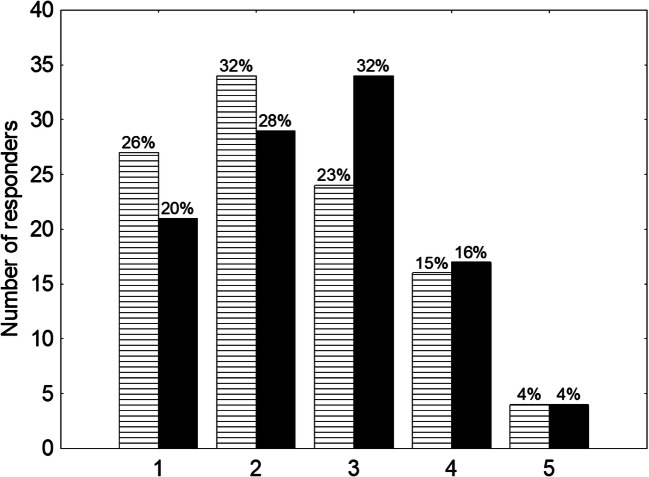
Satisfaction with diagnostic imaging and physics training. A 5-point scale was used to categorize qualitative answers (from 1, which stands for less satisfied to 5, which stands for very satisfied). Black-coloured columns represent answers regarding satisfaction with physics training while white stripes columns represent answers concerning diagnostic imaging training

The quality of diagnostic imaging and physics education were also assessed and 58% (question about diagnostic imaging) and 48% (question about physics) of responders were unsatisfied (1 or 2 points on the Linkert scale) with this aspect of education. Only 19% and 20% stated that the quality of education in diagnostic imaging and physics was satisfactory and scored respectively 4 and 5 points on the Linkert scale (Fig. 3).

The quality of training, courses, educational and clinical cooperation with a supervisor should translate into “good practice” understood as treatment provided according to the best available evidence, including the latest recommendations, guidelines, protocols and delineation atlases. A question concerning such manner (“Do you contour and treat patients according to guidelines/protocols and respect the rule of overall treatment time?”) showed that in the majority (70%) of hospitals, patients are treated according to available guidelines or hospital-specified protocols. However, 30% of participants responded that the principle of “good practice” is often not observed, which is identified as poor access to the latest scientific data, lack of standardized protocols, ignorance of contouring guidelines or refusal to help by more experienced specialists.

2. **Traineeships**

List of mandatory traineeships is presented in Table 3. Two ways of conducting the training are available, based on organization of the hospital (in “organ-specified” or “non-organ specified” way). Traineeships are organized in hospital in which training is held or, if there is no appropriate department, in external hospitals. Traineeships last from one to 24 months. An evaluation of a traineeship should be conducted in a theoretical and practical manner by the traineeship supervisor at the end of the traineeship. However, the form of evaluation is not standardized and at the discretion of the supervisor.

**Table 3:** List of mandatory traineeships

List of internships mandatory in years 2003–2014	List of internships mandatory from 01.10.2014 until now
Variant I 1. Radiotherapy department (24 months) 2. Brachytherapy department (4 months) 3. Radiotherapy clinic department (12 months, including 4 months at gynaecology department) 4. Oncology clinic (8 months) 5. Oncologic surgery department (2 months) 6. Clinical oncology department (3 months) 7. Nuclear medicine department (2 months) 8. Diagnostic imaging department (1 month) 9. Pathology department (1 month) Variant II 1. Radiotherapy department (24 months) 2. Head and neck tumours department (4 months) 3. Brachytherapy department (4 months) 4. Gastrointestinal tumours department (3 months) 5. Haematological malignancies department (3 months) 6. Breast cancer department (3 months) 7. Thoracic neoplasms department (3 months) 8. Gynaecological malignancies department (4 months) 9. Genitourinary tumours department (3 months) 10. Department of neoplasms of soft tissues and bones (2 months) 11. Nuclear medicine department (2 months) 12. Diagnostic imaging department (1 month) 13. Pathology department (1 month)	Variant I 1. Radiotherapy department (24.5 months) 2. Brachytherapy department (4 months) 3. Radiotherapy clinic department (11 months and 1 week, including 4 months at gynaecology department) 4. Oncology clinic (4 months) 5. Oncologic surgery department (1 month) 6. Clinical oncology department (3 months) 7. Nuclear medicine department (1 month) 8. Diagnostic imaging department (2 months) 9. Pathology department (2 weeks) Variant II 1. Radiotherapy department (20 months) 2. Head and neck tumours department (3 months and 3 weeks) 3. Brachytherapy department (3 months and 2 weeks) 4. Gastrointestinal tumours department (3 months) 5. Haematological malignancies department (3 months) 6. Breast cancer department (3 months) 7. Thoracic neoplasms department (3 months) 8. Gynaecological malignancies department (3 months and 2 weeks) 9. Genitourinary tumours department (3 months) 10. Department of neoplasms of soft tissues and bones (2 months) 11. Nuclear medicine department (1 month) 12. Diagnostic imaging department (2 months) 13. Pathology department (2 weeks)

One question regarding the length of traineeships showed that only 33% of responders participated in traineeships according to the programme. Two-thirds (67%) of ROTs responded that traineeships are shorter than planned; in the majority of cases, it is due to lack of medical professionals at their own departments. When asked about the preferred ways of doing a traineeship (multiple-answer question), 34.3% wanted to take part in 1-month traineeships, organized in high-specialty hospitals, 43.8% wanted to do 2-week traineeships in many high-specialty hospitals in Poland, 45.7% wanted to take part in 1-week topic-defined traineeship in different hospitals, 48.6% wanted to participate in master-student classes lasting a few days and only 1.9% were not interested in such a form of education.

3. **Conferences**

Responders were asked to give the type of conferences in which they participated. Of all the participants, 65.7% took part in at least one national conference, 39% participated in international conferences (majority in ESTRO/ESMO meetings) and 24.8% never participated in any national or international meeting. When asked about participation in non-mandatory courses and activities, 53% of ROTs stated that they did not have such an opportunity, mostly due to lack of financial support from the hospital. In one or several educational events per year, 23% of ROTs had an opportunity to participate, and 24% had such possibility less frequently.

4. **Journal clubs**

Our survey contained only one question referring to membership in journal clubs which means regular participation in meetings to present or discuss recent advances in radiation oncology. Although such meetings are mentioned in the teaching programme (as “active participation”), only 11% of responders answered that they have been members of journal club.

5. **Scientific work**

Almost two-thirds of radiation oncology residents (65.7%) work only in clinics, while 34.3% do both clinical and scientific work. Such results have implications in practice: 54.3% never published an article or wrote only one case report/review, obligatory in a specialty programme (but it is not mandatory to publish that in any journal). Of those who do scientific work (multiple-answer question), 9.5% were co-authors of one publication, 5.7% were the first authors of one publication, 18.1% were co-authors of the first authors of 2–3 publications, 10.5% were co-authors of the first authors of more than 3 publications and only 2.9% published more than 10 studies as co-authors of the first authors A PhD degree was obtained during a specialty training only by two ROTs. The major obstacles in scientific work included: lack of knowledge about methodology or statistics (24.8%), lack of scientific cooperation in the hospital or lack of help from more experienced scientists (24.9%), lack of consent from the supervisor (3.8%), lack of time (3%) or lack of concepts (10.5%). Only 3.8% of responders declared that they do not have any difficulties regarding scientific work.

## Discussion

The position of ROTs in radiation oncology in Poland has been poorly documented on the basis of nationwide questionnaires. To the best of our knowledge, this is the second report on that issue in Central/Eastern Europe. The first one, also Polish, was published in the form of a short letter [12]. In this study, we aimed to comprehensively evaluate the quality of radiation oncology training in Poland and to identify difficulties and needs of ROTs. We were able to obtain answers from all administrative regions of our country. However, regional differences can be seen (Table 4). Yet, this is in line with the number of facilities and patients treated in particular parts of Poland [15, 16].

**Table 4:** Comparison of radiotherapy facilities in terms of location, number of residents, number of survey responders and number of patients treated in the year 2018

Voivodeship	Number of radiotherapy facilities	Number and locations of facilities with radiation oncologist in training	Number of radiation oncologist in training in 2018*,**	Number of survey responders among radiation oncologist in training (%)	Number of patient treated in 2018
1.Lower Silesia	3	1 – Wrocław DCO	9	5 (56%)	4921
2.West Pomerania	2	1 – Szczecin	3	3 (100%)	3523
3.Pomerania	2	2 – 1.Gdańsk	4	5 (63%)	2615
		2.Gdynia	4		1790
4.Warmia-Masuria	2	2 – 1.Elbląg	1	3 (60%)	1348
		2. Olsztyn	4		2225
5.Lubusz	2	1 – Zielona Góra	2	1 (50%)	1825
6.Greater Poland	2	1 – Poznań WCO	13	10 (77%)	5704
7.Kujawy-Pomerania	1	1 – Bydgoszcz	7	5 (71%)	6861
8.Masovia	5	1 – Warszawa COI	19	14 (74%)	7288
9.Podlaskie	1	1 – Białystok	10	6 (60%)	2490
10.Łódzkie	2	1 – Łódź	7	7 (100%)	4173
11.Lubelskie	3	2 – 1.Lublin	13	5 (33%)	2532
		2.Zamość	2		1345
12.Opolskie	1	1 – Opole	3	2 (67%)	1095
13.Silesia	6	5 – 1.Bielsko-Biała	2	15/23* (65%)	1718
		2. Częstochowa	1		1015
		3. Gliwice	18		8108
		4. Katowice KCO	4		3620
		5. Katowice CDiTO	2		1425
14.Świętokrzyskie	1	1 – Kielce	1	1 (100%)	2397
15.Lesser Poland	7	4–1.Kraków COI	17	15/20** (75%)	1273
		2. Kraków Amethyst	5		2724
		3. Nowy Sącz	1		528
		4. Tarnów	1		982
16.Subcarpathian	2	2–1.Brzozów	4	7 (88%)	1481
		2. Rzeszów	4		1785

*The number of radiation oncologist in Silesia increased during 2018, from 23 to 27, but 23 were initially analysed and asked to complete the survey

**The number of radiation oncologist in Lesser Poland increased during 2018, from 20 to 24, but 20 were initially analysed and asked to complete the survey

To provide the comparison between facilities we divided them by the number of patients treated per year (small, less than 2 thousand patients per year; medium-sized, 2 to 4 thousand patients per year; and big, more than 4 thousand patients per year). Consequently, we included in the study 25 responders from small radiotherapy departments, 29 from medium-sized and 51 responders from big radiotherapy departments. Less (68%) responders from small hospitals were satisfied with their teaching place compared to medium-sized (83%) and big (86%) facilities. Satisfaction with the choice of radiation oncology as specialty (4 or 5 points on the Linkert scale) was quite similar, i.e. 88% responders came from big departments, 75%, from medium-sized and 88%, from small hospitals. Satisfaction with the quality of radiation oncology training (4 or 5 points on the Linkert scale) was the lowest in small hospitals (28%) compared to 48% in medium-sized and 48% in big hospitals. The number of patients treated per month correlated with the size of the hospital, and only in big radiotherapy departments almost 60% take care of more than 20 patients per month, which is not that common in medium-sized (24%) or small departments (28%). Close or constant clinical cooperation with the supervisor was noted in 77% and 79% of trainees working in big and medium-sized facilities, and slightly less, in 72% of those employed in small hospitals. Good educational cooperation was noted by 48% of responders from big hospitals, in 55% from medium-sized and only in 28% of responders working in small hospitals.

We observed a striking difference between the level of satisfaction with the choice of specialty and with the training (85% vs 43%). This finding suggests that some aspects of the training need refinement and justifies conducting this study. What is surprising is that, in comparison to the previous Polish report, more ROTs are dissatisfied with both the above-mentioned aspects despite having access to better equipment, knowledge and scientific activities as well as being provided with a higher salary or better working conditions.

In terms of the courses provided during the training, we noted that there were several needs often reported by the participants. The subjects we found to be the most commonly reported were evidence-based medicine and basic statistics (*n* = 10), contouring courses (*n* = 26), physics and radiotherapy planning (*n* = 15) and radiographic anatomy (*n* = 18). It clearly shows that the most needed courses were the ones focused on practical aspects of radiotherapy planning and delivery. The reason for such demand may be the fact that about half of the responders were unsatisfied with diagnostic imaging and physics education. Introducing some changes into current courses, rather than introducing new courses would probably help to satisfy responders’ needs and come up to their expectations. The fact that courses which vaguely comprised the subject of radiation oncology, e.g. public health, appeared to be least useful, clearly reveals expectations of trainees.

The analysis of questions regarding traineeships brought worrying results. We found that only one third of the ROTs took part in traineeships which were conducted in compliance with a previously arranged teaching plan. The participants provided suggestions which could help improve the situation, e.g. short exchanges which could be made more often. Some improvements seem highly essential when we bear in mind the fact that due to poor working conditions and remuneration of qualified doctors, the Polish medical system suffers from an insufficient number of medical professionals almost of all specialties. Leaving work in a hospital for 1 month or longer is sometimes very difficult because such action would disturb working conditions in the primary place of employment of ROTs.

One way of gaining the latest knowledge is taking part in national or international conferences, as well as international or online courses. This particularly applies to rapidly developing and very demanding medical specialties such as radiation oncology. At the end of 2018, every doctor taking part in training was provided with extra 6 days off each year. However, more than half of ROTs have no opportunity to participate in such events, mostly due to financial reasons. Lack of financial support was also the most important factor preventing from engaging in additional educational and clinical activities outside a home institution. This area needs urgent improvement. Several methods of external funding (including ESTRO grants, national scholarships) are available; however, they are rarely used by Polish ROTs.

A limitation of this study is a response rate of 68%. This problem was observed in other studies conducted both in Europe and North America [4–11]. The unwillingness to participate in this study can be explained by several reasons. We speculate that lack of interest in the subject and a relatively short period of study time were the main reasons. It should be pointed out that this was the first attempt, made on such a scale in Poland, shortly after formation of youngPTRO structures. However, we admit that a larger response rate would have enabled to identify other demographic or programme-specific factors associated with difficulties and needs of ROTs. Several attempts may have been made to improve the response rate [17, 18]. On the other hand, the literature suggests that these days a response rate can be no longer an evidence to judge study quality and/or validity [17]. Another limitation of the study is lack of additional evaluations made by radiation oncology teachers, radiation oncology specialists and independent experts that might be supplementary for our results. Finally, we mentioned changes in the specialization programme which were introduced in 2014. Since then, two versions of the programme have been available, and this heterogeneity hampers comprehensive evaluation. However, as mentioned in the Results, the majority of responders participated in the “old version”, i.e. the one implemented before 1 October 2014.

We believe that in order to monitor changes in the situation ROTs and to improve data quality, surveys should be conducted repetitively. The future questionnaire should also be made available for completion at the PTRO annual meeting to get more responses, because many ROTs attend this meeting for education purposes. The PTRO, as the leading organization for radiation oncologists in Poland, should also take measures to encourage nationwide surveillance programmes for ROTs training. Future surveys in Poland will enable to evaluate development progressively and improve radiation oncology teaching programme nationwide.

## Conclusions

Radiation oncology training in Poland is in line with the ESTRO recommendations included in the last Core Curriculum. However, it needs many improvements. Introduction of organ-specified courses is an appropriate modification of the previous program. However, this notion needs further validation in subsequent surveys addressed to ROTs who follow the new version of the programme. Noteworthy, diagnostic imaging and physics courses are not sufficient for most participants. Some hospitals need to improve clinical and educational cooperation between trainees and their supervisors. Financial support provided by the hospital is crucial as it enables ROTs to participate in international conferences and additional courses. Constant training quality monitoring is essential to maintain achievements made in previous years, assess current needs and implement necessary changes.

## Electronic supplementary material

ESM 1 (DOCX 16 kb)
