# Supplementary material for: Radiation Oncology Training in Poland: Multi-institutional Survey
Source: J Cancer Educ. 2020 Feb 12;36(4):769–78. doi: 10.1007/s13187-020-01702-8 (PMC8328852; doi:10.1007/s13187-020-01702-8)
Supplement: Supplementary file 1 — (DOCX 16 kb) [file 13187_2020_1702_MOESM1_ESM.docx]

1. I am working in … (the name of the hospital/city)
2. How long are you working?
   - less than a year
   - 1-3 years
   - 3-5 years
3. Are you satisfied with the choice of place where do you specialize?
   - yes
   - no (why?)
4. How many radical / palliative radiotherapy patients per month do you have:
   - less than 10 patients
   - 10 to 20 patients
   - 20 to 30 patients
   - more than 30 patients
5. Do you think that the number of patients you treat is:
   - too low
   - adequate
   - too high
   - the diversity of oncologic cases is unsatisfactory
6. Clinical cooperation with a specialist / internship supervisor / specialization supervisor:
   - constant cooperation, co-shared patients care with the specialist
   - I take care of the patients separately from a specialist but cases and treatment plans are consulted by the supervisor
   - cases and treatment plans are consulted by the supervisor, but cooperation is difficult
   - there is no help from supervisor or other doctors
7. Educational cooperation with a specialist / internship supervisor / specialization supervisor: (select all correct)
   - regular consultations, discussion about the guidelines or latest reports
   - discussion about the guidelines, reference to literature
   - rare conversations about recommendations / studies
   - there is no such cooperation / I have to look for everything myself
   - we have a journal club where the latest reports are discussed
8. Do you work independently in an oncology clinic?
   - yes, from the beginning of the specialization
   - yes, but after finishing the second year of training
   - no
9. Do you work somewhere outside the primary workplace? If so, for what reasons?
   - no
   - yes, for financial reasons
   - yes, because of my interests
   - yes, continuation of previous work
10. Are patients in the center treated in accordance with current guidelines / does the center have treatment protocols for most of the diseases? Are the total treatment time (OTT) rules followed? Do you contour according to the guidelines?
    - yes, in most of the patients the procedure is in line with the guidelines / protocols are updated on an ongoing basis / contouring is done using atlases / literature recommendations, OTT rules are followed
    - the center has protocols from other hospitals, guidelines are followed, contouring based on guidelines from atlases, OTT rules are followed
    - guidelines are available but not followed, contouring according to central protocols, OTT varies
    - it is hard to get access to the guidelines, I draw how specialist taught me, the treatment time does not matter
    - I do not know the current guidelines, it is difficult to get clear contouring recommendations, the treatment time is usually long
11. Do you work:
    - clinically only
    - clinically and scientifically
    - scientifically only
12. Do you think that you spend too much time at work?
    - yes
    - no
13. How many publications do you have during the specialization? How much as the first author? (choose more than one if correct)
    - none or only case description / congress summary required to complete the specialization
    - 1, but I was not the first author
    - 1, I was the first author
    - 2-3 as the first author and the next
    - more than 3 as the first author and the next
    - more than 10 as the first author and the next
    - I did a doctorate during the specialization
14. If you work scientifically, what is the most difficult for you? (select all correct)
    - I don't work scientifically
    - lack of the knowledge about research methodology / statistics
    - lack of the ideas
    - lack of help from experienced researchers / lack of scientific cooperation at the center
    - lack of consent from supervisors
    - I have no difficulties in scientific work
15. What courses do you consider unnecessary during the training?
16. What courses would be recommended?
17. Is specialization training conducted in accordance with the program?
    - yes, traineeships and courses take place according to the program
    - yes, but some traineeships are shorter and I am then in the radiotherapy department / in the radiotherapy ward / in a clinic where is lack of doctors to work
    - rather yes, but the traineeships are shorter and I spend most of my time in the radiotherapy department / in the radiotherapy ward
    - there are problems (if so - what?)
18. Do you have the possibility (financially from the center, not on your own) to take non-obligatory courses during your specialization, e.g. ESTRO courses?
    - yes, one to several per year
    - yes, but this is rare
    - the hospital has no funds for it, it is not possible to do that
    - I don't want to go for such courses
19. During the specialization I had the opportunity to participate at least once (select all that apply to) in:
    - ASTRO
    - ASCO
    - ESTRO
    - ESMO
    - ECCO
    - ESSO
    - Polish Clinical Oncology congress
    - Polish Oncology congress
    - I don't participate in scientific conferences
20. What form of internship would you be interested in?
    - monthly traineeships at the reference center
    - two-week traineeships in several centres in the country
    - weekly topic-based traineeships in various centres
    - a few-day master-student classes
    - I am not interested in this form of education
21. Is the educational platform to exchange knowledge / recommendations / discuss difficult cases:
    - it is a good idea to exchange scientific knowledge, I would use it
    - it is a good idea for exchange of knowledge and scientific cooperation between centres, I would like to co-create it
    - it's a bad idea, it won't work
22. On a scale of 1 to 5, select your satisfaction from (1 = Very bad, 5 = Very good):
    - the specialization itself
    - the quality of training
    - the amount of free time after work
    - the financial aspects of work
    - the future perspectives
    - the radiology training
    - the training in medical physics
    - the cooperation with physicists team
    - the cooperation with technicians team
